# Increase of Adverse Events After Intravenous Injection of Gentamicin in Horses Between 2015 and 2017—From Marketing Authorization Holder's Point of View

**DOI:** 10.3389/fvets.2021.710571

**Published:** 2021-08-16

**Authors:** Viola Stammwitz, Änne Honnens, Dieter Hochhuth, Hans-Joachim Schuberth

**Affiliations:** ^1^CP-Pharma, Burgdorf, Germany; ^2^Institute of Immunology, University of Veterinary Medicine Foundation, Hanover, Germany

**Keywords:** gentamicin, equine, histamine, adverse reaction, impurity, pharmacovigilance, fermentative API, human

## Abstract

Between 2015 and 2017, a marked increase of anaphylactic-like reactions after intravenous administration of gentamicin was observed first in horses and, later, also in humans. This worldwide issue led to safety measures including product recalls and safety warnings. Here, a German Marketing Authorization Holder (MAH) of an early and intensely affected veterinary product containing gentamicin describes the clinical approach of the company to analyze the root cause and identify the causative agent in the active pharmaceutical ingredient (API). The pharmacovigilance data of the MAH are presented, along with pharmacovigilance phenomena observed during the affected period. An overview is given on further investigations of the API manufacturer and measures taken by all parties involved, including competent authorities to reestablish a safe use of gentamicin products. The histamine contamination of gentamicin was an exceptional incident of global extent, affecting not only veterinary but also human drug safety. The reactions in horses transpired to also be an indicator of a human health threat, which ultimately contributed to an improvement in the safety of human and veterinary medicinal products containing fermentative APIs. The extreme dimensions of this issue emphasise the important role that veterinary clinicians and practitioners play in spontaneous reporting based pharmacovigilance systems and, by this, in drug safety.

## Introduction

**Figure 1:**
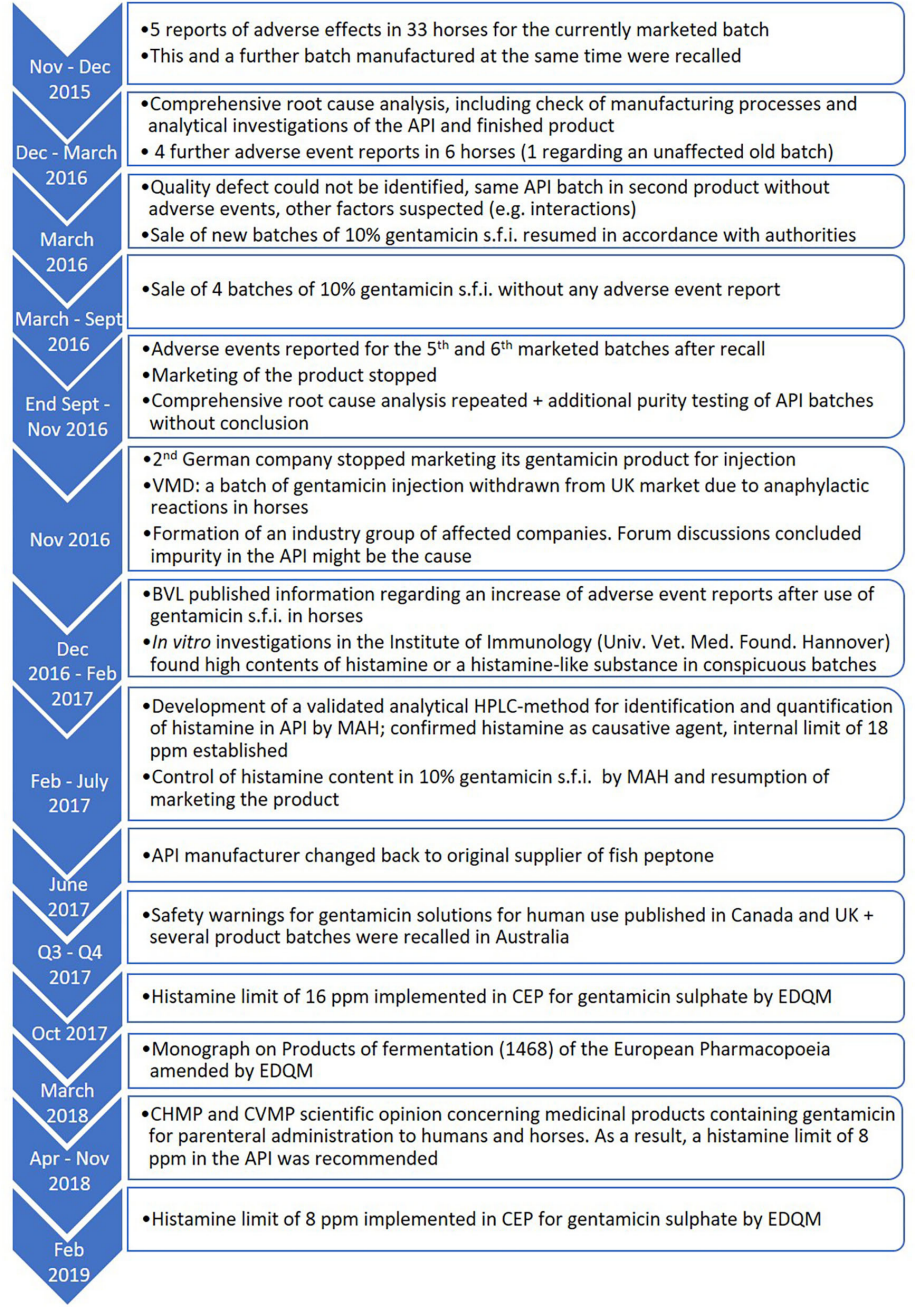
Timeline of events regarding the safety of gentamicin between 2015 and 2019. It should be noted that this figure is not exhaustive and serves for orientation only.

The aminoglycoside, gentamicin, is a widely used antibiotic in equine medicine. As with humans, the preferred administration route for systemic use in horses is *via* intravenous injection. In the EU, the regulatory demands on quality of veterinary medicinal products (VMPs) and their active pharmaceutical ingredients (API) are similar to those of human medicinal products. This means that Good Manufacturing Practice (GMP) standards fully apply, including control of raw materials and manufacturing steps, as well as analysis of every finished product batch prior to market release. As a result of these stringent measures, undetected product defects leading to adverse events are very rare. Veterinary pharmacovigilance is the postmarketing surveillance of product safety, which is predominantly based on spontaneous reports originating from veterinary practitioners. It serves mainly for detection of rare adverse effects, which are too infrequent to be reliably discovered during clinical trials for authorisation. On rare occasions, as occurred in this case, it also serves to recognise quality defects that remain undetected by routine quality control testing. In the following, we present an example of such a rare incident from the point of view of the Marketing Authorization Holder of authorship (referred to as the “MAH”), when between 2015 and 2017, a marked increase of adverse events was observed in horses after intravenous injection of gentamicin. This article describes the details of the pharmacovigilance data of the MAH and their scientific evaluation, as well as the cascade of investigations for root cause analysis and the measures taken to reestablish the safety of the product. The timeline of events, starting with the initial reports of adverse events in 2015 and showing safety measures implemented up to 2019, are shown in Figure 1.

## Course of Events

In November and December of 2015, the German MAH received five adverse event reports in a total of 33 horses after intravenous injection of a batch of its 10% gentamicin solution for injection (Genta 100 mg/ml, CP-Pharma) that was marketed at the time. The reported signs were similar to anaphylactoid reactions. Three of the five reports involving 22 horses were all received on the same day and triggered an immediate recall of the associated batch. As a precautionary measure, an additional batch that was manufactured at the same time as the affected batch, was also recalled. Triggered by the recall, four additional reports of adverse events in six horses were also received by MAH.

Comprehensive root cause analysis, including a check of manufacturing processes and analytical investigations of the API, gentamicin sulphate, and the finished product, 10% gentamicin solution for injection (s.f.i.), remained inconclusive. The analytical investigations of the API were performed according to European Pharmacopoeia. The API manufacturer stated that they had made no changes to the manufacturing process. Routine analysis of the finished product comprised the following parameters: sterility, clarity and colour of solution, visible particles, pH, relative density, identification and quantification of the active substance and excipients, quantification of the gentamicin components, unknown single impurities, and total impurities. These pharmaceutical investigations of the finished product did not identify any parameter that deviated from specifications. Furthermore, additional investigations that are not part of routine analytics (endotoxins, test of abnormal toxicity) provided no suspicious results. The same API batch was used in a second product of another pharmaceutical company (not in Germany), which received no reports of adverse events at that time. Therefore, since a quality defect could not be identified, other factors such as interactions with other substances or veterinary medicinal products leading to an increase of adverse event reports were considered.

In agreement with Competent Authorities, the sale of new batches of 10% gentamicin s.f.i. was resumed in March, 2016. Four batches were sold without receiving any further adverse event reports.

From the end of September to mid-October, 2016, adverse events similar to those from the previous year were again reported for a fifth batch marketed after the recall by two practices in a total of nine horses. In November 2016, further reports were received for a sixth batch, at which point, the MAH decided to stop marketing the product.

A comprehensive root cause analysis was repeated. In addition to the previous pharmaceutical investigations of the batches of finished product and associated API batches, further purity testing of API batches was included. This involved using micellar electrokinetic capillary chromatography (MEKC) (1), an optimised method for separating the components of aminoglycoside antibiotics. These investigations did not reveal significant differences between (a) API batches and the chemical reference substance, or (b) differences between conspicuous API batches used for the finished product causing adverse events and inconspicuous API batches used for finished product not causing adverse events.

As pharmaceutical investigations could not identify any scientific reason for the increase in adverse reactions, already after the recall in 2015, reporting veterinarians were requested by the MAH to provide information, not only on other substances administered to the reacting horse over the same time period, but also on treatments, especially vaccinations, within the previous 12 months. This was conducted to check for possible unknown interactions that might trigger the reactions; however, the evidence did not support this possibility. Another consideration was whether the fact the reactions started again in autumn was relevant or if the timing was just a coincidence. It seemed most probable that an impurity in the API caused the reactions but, if so, other marketing authorisation holders should have experienced the same problems, as at that time only two API manufacturers worldwide produced gentamicin of a quality acceptable for European and some overseas markets.

Further developments occurred in November 2016, when another German pharmaceutical company stopped the marketing of its gentamicin product for injection. At the same time, the MAH recognised that the Veterinary Medicines Directorate (VMD, UK) published information that a batch of gentamicin injection was withdrawn from the UK market due to serious adverse events resulting in anaphylactic reactions in horses. As contact details of the pharmaceutical company involved in the UK were also published, it was possible to share information with them. Several other European pharmaceutical companies affected by this issue also joined the discussion. The exchange of information between these independent pharmaceutical companies supported the suspicion that an impurity in the API might be the root cause of the adverse reactions in horses because all gentamicin injections involved contained API from the same API manufacturer.

The API, gentamicin sulphate, is produced by fermentation and consists of a mixture of different chemical entities, four major (named C1, C1a, C2, and C2a) and several minor components, making analytical investigations challenging. Since no unusual impurities could be found with routine and even advanced analytical investigations, an approach from the clinical point of view was pursued.

## Adverse Events

The reports stated that the horses affected showed signs of generalised hypersensitivity reactions, some of which occurred already during the injection but, mostly, immediately after the injection was complete. In mild cases, the horses only showed signs such as trembling, tachycardia, increased respiratory rate, and restlessness. Notably, the majority of horses exhibited signs of colic of varying intensity, such as pawing, lying down (more or less vigorous), rolling, flehmen, and sweating. A summary of the clinical observations using VeDDRA (2) is shown in Table 1. Additional observations included various vocalisation, such as snorting or groaning and, on occasion, increased frequency of defecation. Coughing was reported in three instances. Some horses had circulatory disorders but only one horse collapsed. In most cases, the signs lasted for a few minutes (up to 10–15 min) and disappeared without treatment. In 29 of 115 horses reacting after the administration of conspicuous batches, the reaction lasted longer than 15 min. At least 18 of these 115 horses were treated with dexamethasone, while it was deemed necessary to treat the colic symptoms of six horses, and in two cases, fluid was administered.

**Table 1:** Count of clinical signs reported as adverse events coded by VeDDRA terminology (2) (SOC, system organ class; PT, preferred term; LLT, low-level term) of conspicuous batches.

**Species**	**Clinical sign VeDDRA SOC**	**Clinical sign VeDDRA PT**	**Clinical sign VeDDRA LLT**	**No. of reports**	**No. of affected animals**
Horse	Behavioural disorders	Behavioural disorder NOS	Rolling	8	10
		Hyperactivity	Agitation	2	4
			Restlessness	21	39
		Vocalisation	Groaning	7	8
			SOC total	**31**	**54**
	Cardiovascular system disorders	Circulatory disorder NOS	Circulatory disorder NOS	1	8
		Hypotension	Prolonged capillary refill time	1	7
		Tachycardia	Increased heart rate	6	16
			Rapid pulse rate	1	1
			Tachycardia	10	32
			SOC total	**17**	**49**
	Digestive tract disorders	Abdominal pain	Colic	27	46
			Tense abdomen	1	3
		Digestive tract disorder NOS	Digestive tract disorder NOS	5	5
			SOC total	**30**	**51**
	Neurological disorders	Ataxia	Ataxia	2	6
			Falling	1	1
			Staggering	2	2
			Unsteady gait	1	1
		Convulsion	Convulsion	1	1
		Muscle tremor	Hiccup	1	1
			Muscle tremor	5	10
			Shivering	17	38
			Trembling	1	6
		Sensory abnormality	Flehmen response	9	16
			SOC total	**30**	**61**
	Respiratory tract disorders	Cough	Cough	3	3
		Dyspnoea	Dyspnoea	2	8
		Tachypnoea	Hyperventilation	1	1
			Increased respiratory rate	7	18
			Tachypnoea	5	24
			SOC total	**17**	**49**
	Systemic disorders	Anorexia	Inappetence	3	3
		Discomfort NOS	Pawing	24	41
		Lethargy	Reduced responses	2	2
			Weakness	1	1
		Pale mucous membrane	Pale mucous membrane	1	1
		Recumbency	Lateral recumbency	3	8
			Recumbency	3	3
			Sternoabdominal recumbency	1	3
		Systemic disorder NOS	Shaking	3	6
			SOC total	**29**	**46**
	Skin and appendages disorders	Hyperhidrosis	Excessive sweating	1	1
			Heavy sweating	4	7
			Increased sweating	13	27
			SOC total	**18**	**35**
	Immune system disorders	Anaphylaxis	Anaphylactic shock	1	3
		Urticaria	Urticaria	2	2
			SOC total	**3**	**5**
	Application site disorders	Injection site haemorrhage	Injection site bleeding	1	1
			Injection site haematoma	1	1
			SOC total	**1**	**1**
	Uncoded signs	Uncoded sign	Uncoded sign^*^	2	16
			SOC total	**2**	**16**

* *Uncoded sign stands for lying down (10 affected horses) and sawhorse position (six affected horses)*.

One horse under general anesthesia developed tachycardia of 120 bpm as a single observation immediately following injection of the gentamicin product for 2–3 min, followed by a normal heart rate without any treatment. This case indicated a direct influence on the cardiocirculatory system, as the heart rate could not have been affected by pain or fear.

The MAH received information, particularly from larger equine hospitals, that not all horses treated with the same conspicuous batch showed adverse signs. Some of the horses with adverse reactions showed clinical signs not before the second or third day of treatment. It was unclear whether in all of these cases the same batch was used on the treatment days before reactions occurred. At least one horse undoubtedly received three treatments with a conspicuous batch before reacting after the fourth dose.

In all reacting horses, the 10% gentamicin s.f.i. was administered intravenously as a bolus. It was also stated by several reporting veterinarians that injection was carried out slowly.

No reports of anaphylactic-like reactions were received for other species. In addition to its use in horses, the product is labeled for use in cattle, pigs and small animals. However, due to very long withdrawal times or better alternatives for other species, this product is mainly used in horses. Furthermore, other administration routes such as subcutaneous and intramuscular injections are possible in other species.

**Figure 2:**
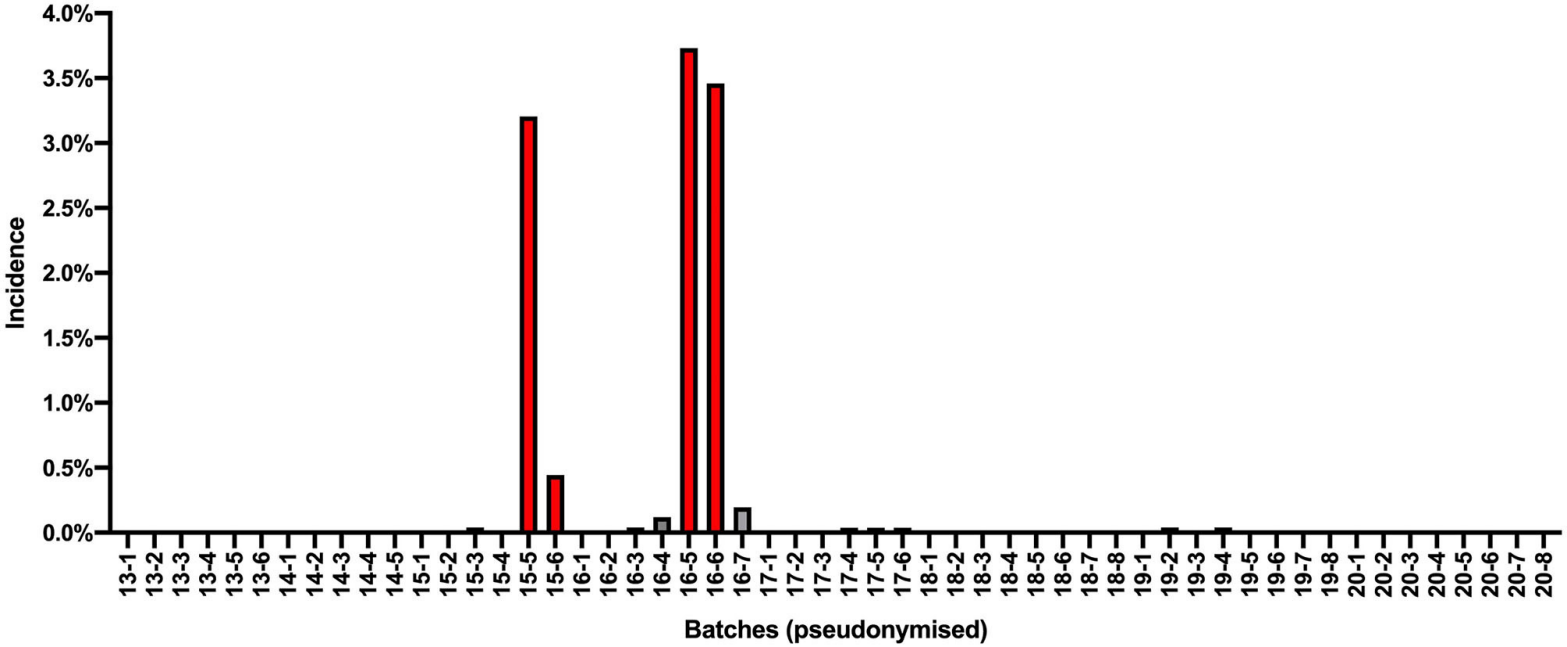
Incidence of adverse events in horses (number of reacting horses per calculated number of treated horses in %) received 2013–2020 displayed per batch. Batch numbers are pseudonymised. Incidences of conspicuous batches are highlighted in red. Sales volumes of these batches are lower due to safety measures (recall, marketing stop). Incidences of inconspicuous batches are denoted in blue. *Batch 15–6: sales volume was very low due to recall together with batch 15–5. In one report, the batch number is unknown, this case is not included. In four adverse event reports of five horses, it was unclear which of the two batches (16–5 and 16–6) was used; thus, for incidence calculation, three horses were assigned to 16–5 and two were assigned to 16–6. Batches 15–3, 16–4, and 16–7: uncertainty if stated batch number is correct in one horse each. In one report, the horse received batch 19–2 mixed with batch 18–4; for incidence calculation, the case was assigned to batch 19–2*.

As a result of the thorough communication during collection of adverse event reports, most cases could be assigned to finished product batches. Figure 2 shows that some batches of 10% gentamicin s.f.i. were linked to an increased incidence of hypersensitivity-like adverse events (referred to here as “conspicuous batches”). For these batches, safety measures (recall, marketing stop) were deemed necessary. In inconspicuous batches this pronounced increase in number of reports of hypersensitivity-like adverse events was not observed and, for these, safety measures were not necessary.

In this context, it is important to understand that anaphylactic reactions can be expected in the usual safety profile of gentamicin solutions for injection. Therefore, this type of adverse event alone might not be sufficient to clearly distinguish between a reaction that can be expected and a reaction that occurred due to an additional unusual factor. As a result, the number of reports and diligent assignment to batches played a major role in the interpretation of the data. Figure 2 reflects all adverse events in horses received between January 2013 and December 2020, but available data were, of course, not as comprehensive at earlier time points.

## The Clue

As mentioned above, due to the heterogenous character of the API, it was difficult to find an impurity that might have caused the adverse reactions. In the 2000s, one group investigated the quality of gentamicin API samples after the administration of a once daily dose of gentamicin caused the death of ~60 people in the USA in 2000 (3). They found sisomycin to be a marker substance for API samples with a high impurity content. The MAH investigated conspicuous and inconspicuous API batches using MEKC (1) analysis, but neither the sisomycin content nor the impurity profile were suspect in any of the samples.

As analytical methods seemed to be exhausted, the idea developed to look from the clinical point of view to better understand what happened in the reacting horses. Some horses showed clinical signs and some did not; therefore, a hypothesis was proposed that these reactions reflect allergic/allergoid responses. In order to determine whether gentamicin s.f.i. triggers the release of histamine, the MAH initiated investigations of blood samples from “reacting” to “nonreacting” horses, which were provided by committed reporting veterinarians. The Institute of Immunology of the University of Veterinary Medicine, Foundation, Hannover (Tierärztliche Hochschule Hannover) investigated whether the gentamicin product led to immunological processes (histamine release of basophilic granulocytes) after incubation with blood *in vitro*. To do this, the equine blood samples (of reacting and nonreacting horses) were incubated with different concentrations of the finished product (10% gentamicin s.f.i.) that included conspicuous as well as inconspicuous product batches. Placebo (finished product solution without gentamicin) was tested in parallel. The incubation was followed by histamine detection in the cell-free supernatant using a radioimmunoassay (RIA) (4, 5). Very low concentrations of histamine below 1 ng/ml can be detected by this method. This test is used for the diagnosis of allergen sensitization in horses. If the product solutions would have triggered a release of inflammatory mediators from basophils, histamine would have been detected in the supernatant. Surprisingly, the results of this test showed that some gentamicin s.f.i. batches themselves contained significant levels of histamine. These batches were identified to be the conspicuous batches with an increased frequency of adverse events. The tested inconspicuous batches also contained histamine but only in very low levels. The placebo (finished product solution without gentamicin) did not contain any histamine. Thus, it became very likely that the cause for the clinical reactions was a contamination of the API with either histamine itself or a histamine-like substance cross-reacting with the antibody used for the RIA (in detail for precipitation of acylated histamine).

The clinical signs observed in reacting horses could be readily explained by the pathophysiological effects of histamine, including vasodilation with subsequent systemic hypotonia, increasing heart rate and contraction of the smooth muscles in the respiratory and intestinal tract (6). The latter would explain the frequently observed colic signs. A study in humans, in which increasing dosages of histamine were infused intravenously, reported a half-life of histamine of 3–6 min (7). This could account for the timeline of the reactions observed in most of the reacting horses, whereby the duration was no longer than 10 min. The same study also found that atopic subjects with a history of asthma, eczema, or rhinitis and subjects with urticaria tolerated histamine only poorly and exhibited signs with lower dosages than control group patients (7). A variable tolerance of histamine exposure was also recognised in a study in ponies, which showed that ponies with a history of heaves were hyperreactive to IV histamine during acute airway obstruction (8). Histamine inhalation is used as a method to diagnose bronchial hyperreactivity of asthma patients in humans and equids (9, 10). All this would explain the variability of reactions seen in horses ranging from no reaction to severe reactions necessitating treatment.

To determine whether the substance found was histamine itself or another histamine-like substance and to establish a validated analytical method, the MAH initiated the development of a high-performance liquid chromatography with postcolumn derivatization and fluorescence detection (HPLC-FLD) method for the detection and quantification of histamine in the API. This was very challenging due to the heterogenous character of the API and the very low content of histamine (or histamine-like substance) present; nevertheless, a method was established and histamine itself was identified in the API. The quantities of histamine detected by this validated HPLC-FLD method correlated well with the results from the RIA, and it was confirmed that the content of histamine was significantly higher in conspicuous batches than in inconspicuous batches. Based on this HPLC-FLD method and on its own pharmacovigilance data, the MAH established an internal limit of 18 parts per million (ppm) of histamine for analysis of API used for further production of finished product (10% gentamicin s.f.i.). This short-term risk management measure made sure that only products with low histamine levels were placed on the market, ensuring that a safe use in horses was reestablished. The API used for conspicuous batches contained more than 60 ppm histamine. The calculated dose applied to horses (based on a dose of 6.6 mg gentamicin/kg body weight) was 0.5 μg histamine/kg body weight or higher for conspicuous batches. The information on this causative agent was shared with the API manufacturer, Competent Authorities and other concerned pharmaceutical companies.

## A Worldwide Problem

The API manufacturer of gentamicin sulfate started to investigate the root cause of the contamination of the API as soon as it became clear that histamine or a histamine-like substance might be the cause for the increased adverse events. The gentamicin sulfate manufacturing process involves growing gentamicin-producing bacteria with nutrient-rich growth medium in fermentation broths, followed by filtration, separation, and purification steps. Possible sources for the histamine that were considered included an introduction of histamine *via* raw materials, as well as the formation and accumulation of histamine in the fermentation or downstream manufacturing processes. This investigation revealed that fish peptone used as a nutrient for the growth medium was the source of histamine. The API manufacturer had changed their fish peptone supplier in 2014 to a source that had inadequately stored the fish prior to production of fish peptone, resulting in a greater level of fish decomposition. During decomposition of fish, bacterial decarboxylases catalyze the formation of histamine from the amino acid, histidine, which naturally occurs in fish. In short, the elevated histamine levels in the gentamicin were linked to poor-quality fish peptone, caused by improper handling and storage conditions. The API manufacturer switched back to their previous supplier of fish peptone from June 2017 and applied procedures to control histamine levels, which resulted in the majority of API batches manufactured up to mid-2018 with histamine levels between 3 and 8 ppm (11, 12).

The API manufacturer is one of only two API manufacturers holding a Certificate of Suitability (CEP) for gentamicin sulfate. The CEP issued by the European Directorate for the Quality of Medicines (EDQM) confirms that the quality of an API complies with the quality described in the relevant monograph of the European Pharmacopoeia, and with this, the API is allowed to be used for finished products—not only for veterinary but also for human products—in the EU. CEPs are also recognised by other countries including, among others, Australia, Canada, South Africa, and Switzerland. Gentamicin sulphate produced by this API manufacturer is also accepted in the USA and Japan. Therefore, as the API manufacturer was one of only two worldwide meeting the high standards recognised by the EU and other countries, the contamination of this API not only concerned horses in the EU but also humans worldwide.

Starting with the adverse events in horses reported to the German MAH, leading to a recall at the end of 2015, an increase in the number of adverse events related to further gentamicin-containing solutions for injections in horses was observed worldwide and, later on, also in humans. The main adverse events observed in humans within the EU were decreased blood pressure and allergic reactions, including one fatality in Italy (11). As far as the authors can ascertain, in the second half of 2017, safety warnings for gentamicin solutions for human use were published in Canada and UK (13, 14) and, in Australia, several product batches were recalled (15). As a short-term risk management measure, the European Directorate for the Quality of Medicines and Healthcare (EDQM) included an interim histamine limit of 16 ppm into the relevant certificate of suitability (CEP) for gentamicin. The limit of 16 ppm was set by the API manufacturer based on their analytical high-performance liquid chromatography with mass spectrometry (HPLC-MS) method for histamine detection. This method found slightly lower histamine levels than the HPLC-FLD method of MAH, but both methods correlated well.

In April 2018, the Executive Director of the European Medicines Agency (EMA) requested the Committee for Medicinal Products for Human Use (CHMP) and the Committee for Medicinal Products for Veterinary Use (CVMP) to offer a scientific opinion concerning medicinal products containing gentamicin for parenteral administration to humans and horses. As a result, a histamine limit of 8 ppm in the API was recommended (11, 12). In February 2019, the histamine limit of 8 ppm was implemented in the relevant CEP of gentamicin sulphate.

Since gentamicin sulfate is not the only API that is manufactured by fermentation processes using fish peptone, other substances may also be at risk of containing elevated histamine levels that could lead to adverse reactions in patients. As a result of a new awareness of this risk, the monograph on Products of fermentation (1,468) of the European Pharmacopoeia was also amended in March 2018.

## Pharmacovigilance Phenomena

Veterinary pharmacovigilance is the postmarketing surveillance of product safety, which is predominantly based on spontaneous reports originating from veterinary practitioners or animal owners. It serves mainly for the detection of rare adverse effects, which are too rare to be reliably discovered during clinical trials for authorisation. For old products with well-known pharmacological and toxicological profiles, interactions with new drugs or new genetics might be detected, or new ways of drug use might change the known profile. Pharmacovigilance also serves to detect quality defects and, due to the high standards in quality control of veterinary medicinal products, adverse events related to these are very rare. A worldwide scenario concerning not only veterinary but also human medicinal products, as exemplified by the histamine contamination of gentamicin, is exceptional. Nevertheless, the pharmacovigilance phenomena observed in this context might also apply for other scenarios.

It is generally known that underreporting is the major limitation of the spontaneous reporting based pharmacovigilance system. This is clearly seen for the 10% gentamicin s.f.i. of the MAH, which is an older product and well-known by equine practitioners. During the 3 years prior to the first report of a reaction due to histamine contamination in November 2015, not a single adverse event was received for the product.

In all five reports received by end of 2015, which led to a recall, more than one horse was affected (three, five, seven, eight, and 10 horses). This reporting pattern demonstrates that veterinarians tended to wait for more than one adverse event before contacting the pharmaceutical company, which was also evident when problems with two further batches started in autumn 2016. Nine out of 10 reports received between September and the end of November, 2016, concerned more than one horse. As the signs observed in affected horses appeared to be hypersensitivity reactions with a drug known to have this as a possible side effect, one mildly reacting horse might have not been remarkable for horse practitioners, who consider a hypersensitivity reaction after intravenous injections of any drug to be a possible scenario. When more horses showed adverse signs, the motivation to contact the pharmaceutical company was greater. To avoid any misunderstanding, it should be noted that a report of adverse events in more than one animal is not indicative of a quality defect. However, it is plausible that from the reporter's point of view, the suspicion of a quality defect becomes more likely when more than one animal shows signs, even though a quality defect is in the vast majority of cases not the cause.

Some veterinarians stated that they wanted to be sure that the gentamicin injection, not concomitant drugs, caused the adverse signs before they made a report. This phenomenon of a degree of bias of veterinarians in reporting adverse events they consider to probably be attributed to the product is already described in literature (16).

As veterinarians became more aware of possible adverse events, this factor might have influenced the reporting behaviour. In November 2016, not only the MAH of authorship but also another German company stopped the marketing of its gentamicin product for injection, and information letters were sent to customers. The German Competent Authority (BVL) published information regarding an increase of adverse event reports after the use of gentamicin injections in horses on its website, as well as in the Journal of the German Veterinary Association (Deutsches Tierärzteblatt) in December 2016 and January 2017. The increase of adverse events in relation to gentamicin was also discussed in international veterinary Internet forums.

It is described for human pharmacovigilance that media attention and publicity resulting from regulatory actions may result in increased reporting, a phenomenon called “notoriety bias” (17, 18). In veterinary pharmacovigilance, this effect is referred to as a “bandwagon effect” (16). It diminishes underreporting by triggering reporting of adverse events that would not be reported under normal circumstances. This may lead to less distinct data by increasing the noise level (18, 19).

This effect could be seen for gentamicin: when implementing safety measures in the market and raising attention to this safety issue, wider reporting of adverse events for inconspicuous batches occurred (see Figure 2).

Triggered by the recall in 2015, which impacted about 230 veterinary practices, four additional reports were received. One report was regarding an already sold-out inconspicuous batch that affected one horse. The other three reports were related to the recalled batches—one included three horses and the other two reports each described effects in one horse.

Starting in December 2016, further cases involving inconspicuous batches were reported. These all included a single horse being affected, except one report with two horses, which received concomitant, possibly causative, drugs. In retrospect, we know that the histamine levels of these inconspicuous batches were as low as those present before the relevant contamination occurred. Prior to the introduction of the new peptone supplier at the API manufacturer in June 2014, levels of histamine were typically <3–12 ppm (12). The API batches used for the inconspicuous 10% gentamicin s.f.i. batches for which adverse events were reported were below 10 ppm, and from 2017 forward, they were below 8 ppm. In December 2019, a clinic that had already experienced some adverse events with conspicuous batches in 2016 reported an adverse event in a horse with a typical hypersensitivity reaction for batch 19–4 (see Figure 2). The histamine level of the API used for this batch was 1 ppm. This suggests that the adverse events in which the inconspicuous batches were involved represent, at least in part, the “normal” adverse event profile of the product, which is not reported under common circumstances. However, it should also be considered that in some affected horses, other drugs with equal causative potential were administered at the same time. It is also interesting to note that these adverse events occurred during a time period in which highly contaminated batches were on the market, which could indicate that the batch involved was incorrectly reported. Indeed, in at least three cases regarding inconspicuous batches there remained some uncertainty as to whether the stated batch was correct, since at the same time, confirmed conspicuous batches were also in use in the reporting practices. In five cases, signs of hypersensitivity presented a different reaction pattern from those reported for conspicuous batches, and/or concurrent drugs capable of causing hypersensitivity were co-administered. In addition, there were reports of one case of renal failure in a horse and one case of renal failure in a dog (not described in Figure 2).

With hindsight, the collection of all available data and knowledge of the root cause of the adverse events, enables a clear batch association and a plausible interpretation of the data. However, at the time of the investigations, the noise occurring due to notoriety bias or a bandwagon effect made the process of classifying, placing, and evaluating the adverse event data for root cause analysis more difficult. Phenomena like these, when triggers lead to a shift in reporting motivation, as well as underreporting, are inherent to the spontaneous reporting-based pharmacovigilance system. It may be helpful for both the reporting veterinarians and the veterinarians managing the adverse event data to be aware of the existence of these phenomena.

## Conclusion

The histamine contamination of gentamicin is an exceptional incident of global extent, affecting not only veterinary but also human drug safety. To put it bluntly, the reacting horse in the stable turned out to be an indicator not only for an equine but also for a human health threat and ultimately contributed to an improvement of the safety of human and veterinary medicinal products containing fermentative APIs. The dimensions of this issue are certainly extreme, nevertheless, it is a good example to emphasise the important role veterinary clinicians and practitioners play in spontaneous reporting based pharmacovigilance systems and by this, in drug safety.

Pharmaceutical companies are still the party collecting the vast majority of spontaneous reports, since they have a vital interest in the safety of their products. They are also in the position for focussed investigations, as all relevant information of their products converge at the marketing authorisation holders. The quality of spontaneous adverse event reports is very important in this context, as high-quality reports have greater value. Therefore, a commitment from both the reporter and the report receiver to due diligence, attention to detail, and devoting the necessary time to generate accurate reports is required. The MAH is thankful that most of the reporting veterinarians exhibited these characteristics and in so doing, contributed to the clarification of this issue.

## Data Availability Statement

The original contributions generated for the study are included in the article/supplementary material, further inquiries can be directed to the corresponding author/s.

## Author Contributions

VS, ÄH, DH, and H-JS: methodology and investigation. VS and ÄH: data collection and validation. VS and H-JS: data analysis and result interpretation. VS: conception and preparation of the manuscript. All authors contributed to manuscript revision, read, and approved the submitted version.

## Conflict of Interest

VS, ÄH, and DH are employed by the company CP-Pharma. H-JS is employed by the Institute of Immunology of the University of Veterinary Medicine, Foundation, Hannover, which was paid by CP-Pharma for the performance of the described Immunoassays.

## Publisher's Note

All claims expressed in this article are solely those of the authors and do not necessarily represent those of their affiliated organizations, or those of the publisher, the editors and the reviewers. Any product that may be evaluated in this article, or claim that may be made by its manufacturer, is not guaranteed or endorsed by the publisher.
